# Dopaminergic modulation of default mode network brain functional connectivity in attention deficit hyperactivity disorder

**DOI:** 10.1002/brb3.582

**Published:** 2016-09-28

**Authors:** Richard B. Silberstein, Andrew Pipingas, Maree Farrow, Florence Levy, Con K. Stough

**Affiliations:** ^1^ Centre for Human PsychopharmacologySwinburne UniversityHawthornVic.Australia; ^2^ Neuro‐Insight Pty LtdMelbourneVic.Australia; ^3^ Wicking Dementia Research and Education CentreUniversity of TasmaniaHobartAustralia; ^4^ HeadChild and Family EastPrince of Wales Hospital and School of PsychiatryUniversity of New South WalesSydneyNSWAustralia

**Keywords:** brain functional connectivity, default mode network, dopamine, methylphenidate, steady‐state visually evoked potential

## Abstract

**Introduction:**

Recent evidence suggests that attention deficit hyperactivity disorder (ADHD) is associated with a range of brain functional connectivity abnormalities, with one of the most prominent being reduced inhibition of the default mode network (DMN) while performing a cognitive task. In this study, we examine the effects of a methylphenidate dose on brain functional connectivity in boys diagnosed with ADHD while they performed a cognitive task.

**Method:**

Brain functional connectivity was estimated using steady‐state visual evoked potential partial coherence before and 90 min after the administration of a methylphenidate dose to 42 stimulant drug‐naïve boys newly diagnosed with ADHD while they performed the A‐X version of the continuous performance task (CPT A‐X).

**Results:**

Methylphenidate robustly reversed the transient functional connectivity increase in the A‐X interval seen premedication to a postmedication decrease during this interval. In addition, methylphenidate‐induced reductions in individual reaction time were correlated with corresponding reductions in functional connectivity.

**Conclusion:**

These findings suggest that methylphenidate suppresses the increased functional connectivity observed in ADHD and that such suppression is associated with improved performance. Our findings support the suggestion that the increased functional connectivity we have observed in ADHD is associated with abnormal DMN activity. In addition, we comment on the significance of specific frequency channels mediating top‐down communication within the cortex and the extent to which our findings are selectively sensitive to top‐down intracortical communication.

## 1 Introduction

Attention deficit hyperactivity disorder (ADHD) is one of the most commonly diagnosed pediatric neuropsychiatric disorders and is estimated to affect up to 6% of children (Brown & Cooke, 1994). In broad terms, ADHD is diagnosed as either comprising predominantly attention deficits or hyperactivity or a combination of both (Levy, Hay, McStephen Wood, & Waldman, 1997). Much of the research into ADHD has tended to focus on executive and motivational dysfunction (Willcutt, Doyle, Nigg, Faraone, & Pennington, 2005). Pathophysiologically, this has been reflected in a focus on prefrontal‐striatal and mesolimbic systems (Castellanos, Sonuga‐Barke, Milham, & Tannock, 2006). An important component of various theories concerning the causes of ADHD has been the role of dopamine (DA). In particular, ADHD was thought to be a consequence of reduced DA activity due to either increased DA synaptic reuptake by or reduced postsynaptic sensitivity at fronto‐striato‐cerebellar networks (Del Campo, Chamberlain, Sahakian, & Robbins, 2011; Tomasi et al., 2009). Over the last decades, significant progress has been made in understanding how ADHD symptoms are related to dysfunction in various components of fronto‐striatal circuitry (dorsolateral prefrontal cortex, DLPFC, ventrolateral prefrontal cortex, VLPFC, dorsal anterior cingulate cortex dACC, and striatum), as well as the parietal cortex, brainstem, and cerebellum (Bush, Valera, & Seidman, 2005; Valera, Faraone, Murray, & Seidman, 2007).

More recently, the focus of ADHD research has shifted to a consideration of ADHD as a disorder of functional connectivity rather than an abnormality restricted to specific cortical regions (Castellanos et al., 2006). An important factor driving this reappraisal is the recognition of the role of a specific cortical network known as the “default mode network” (DMN). The DMN, first reported by Raichle et al. (2001), was identified when examining resting state functional connectivity using fMRI. The DMN is a network comprising a number of regions including the ventrolateral and ventromedial prefrontal cortex, the posterior cingulate cortex (PCC), the cuneus, and the inferior parietal lobe (Buckner, Andrews‐Hanna, & Schacter, 2008). The DMN is most active when awake subjects are resting and not engaged in a cognitive task (Buckner et al., 2008; Raichle et al., 2001). In general, the DMN becomes less active during a cognitive task when other task‐positive networks become in turn more active (see Gerlach, Spreng, Gilmore, & Schacter, 2011; Gerlach, Spreng, Madore, & Schacter, 2014).

Lapses in sustained attention are associated with DMN activity during attentional tasks (Weissman, Roberts, Visscher, & Woldorf, 2006). A reduced negative correlation between the DMN and task active networks has been reported in ADHD (Castellanos et al., 2008; Christakou et al., 2013; Liston, Cohen, Teslovich, Levenson, & Casey, 2011; Sun et al., 2012). Sonuga‐Barke and Castellanos (2007) suggest that the inattentiveness observed in ADHD could be due to inadequate suppression of the DMN and its increased activity is associated with the intrusion of thoughts unrelated to the task or “day dreaming”(Fassbender et al., 2009; Kucyi & Davis, 2014). A meta‐analysis of 55 ADHD fMRI task‐based studies (39 children studies) indicated that the most consistent findings were that compared to controls, the ADHD groups exhibited hyperactivity in the DMN and hypoactivity in the task‐positive networks such as the frontoparietal and ventral attentional networks during cognitive tasks (Cortese et al., 2012).

Increased DMN activity in an attention task is also associated with slower and more variable responses (Buckner et al., 2008). This was observed in an fMRI study by Weissman et al. (2006) where brain activity was observed while participants performed a local/global selective attention task. They found that longer reaction times were associated with reduced prestimulus activity in executive networks involving the anterior cingulate cortex and reduced inhibition or greater activity in the DMN, including the PCC and the precuneus. These observations were confirmed in a study by Prado and Weissman (2011) who reported that a positive correlation between the PCC (a key DMN region) and the DLPC, (a key task‐positive region) was associated with a slower current response in a selective visual attention task. Consistent with the positive correlation of DMN activity and longer reaction times are the observations that daydreaming and task‐independent thoughts are associated with greater DMN activity (Mason et al., 2007) and that response time variability is greater when the inhibitory effect of the task‐positive attentional network on the DMN is weaker (Kelly, Uddin, Biswal, Castellanos, & Milham, 2008).

Further evidence for the role of the DMN in ADHD symptomatology comes from studies examining the effects of increased DA activity on the DMN. In an fMRI study, Peterson et al. (2009) examined the brain activity in the ventral anterior cingulate and posterior cingulate cortices while youths diagnosed with ADHD and controls undertook a Stroop Colour and Word Task. The brain activity was measured twice in youths diagnosed with ADHD, on and off stimulant medication. The study reported greater suppression of the above‐mentioned DMN nodes when youths were on stimulant medication compared to off stimulant medication. Liddle et al. (2011) reported similar findings in a study examining the effects of methylphenidate (MPH), a dopamine reuptake blocker on DMN activity in children diagnosed with ADHD. In this study, children were required to undertake a paced Go/No‐go task when either off or on MPH. Compared to the off MPH condition, the DMN was more strongly suppressed in the on MPH condition. While MPH modulated DMN task‐related inhibition, increased motivation had a similar effect to MPH. This is not surprising given the well‐known effects of motivation on DA activity (Rubia et al., 2009). Further evidence implicating the DMN in ADHD was presented in a PET study examining the relationship between the DA reuptake transporters (DAT) and DMN activity in a visual attention task (Tomasi et al., 2009). The study reported that higher DAT levels, and hence reduced extracellular DA, was associated with reduced DMN suppression in a visual attention task. In summary, the enhancement of task related suppression of the DMN by stimulant medication is consistent with suggestions of inadequate DMN suppression as a causative factor mediating attention deficits in ADHD.

In an earlier study, we reported significant differences in brain *functional connectivity* (FC) in a group of stimulant drug‐naive boys, newly diagnosed with ADHD compared to a control group (Silberstein et al., 2016). Here, FC was assessed using an evoked potential methodology that utilized the steady‐state visual evoked potential (SSVEP) in response to a diffuse flicker (see Silberstein, Cadusch, Nield, Pipingas, & Simpson, 1996; Silberstein, Nunez, Pipingas, Harris, & Danieli, 2001). The evoked potential methodology used to measure functional connectivity makes use of the SSVEP in response to a diffuse flicker (see Silberstein et al., 1996, 2001). We have previously shown that cognitive tasks performed while subjects are simultaneously exposed to an ongoing peripheral spatially diffuse 13 Hz visual flicker are associated with task‐dependent changes in the amplitude and phase of the 13 Hz sinusoidal evoked potential or 13 Hz SSVEP (Silberstein, 1995; Silberstein et al., 1998). The methodology, termed Steady State Topography (and in some earlier papers Steady State Probe Topography) has been used to examine the scalp topography of SSVEP amplitude and phase variations associated with a range of cognitive tasks in typical and patient populations (Silberstein, Line, Pipingas, Copolov, & Harris, 2000; Silberstein et al., 2001). One important advantage of Steady State Topography is the high SSVEP signal to noise ratio is in turn associated with a high resistance to most common electroencephalography (EEG) artifacts such as EOG, blink and movement artifacts, mains interference, and EMG (Gray, Kemp, Silberstein, & Nathan, 2003; Silberstein, 1995).

The methodology used in this study, termed the *SSVEP Event‐Related Partial Coherence* (SSVEP‐ERPC) can provide a measure of the degree to which phase differences between electrode pairs remain stable across trials once the common contribution from the SSVEP stimulus has been removed (Silberstein, 2006; Silberstein, Song, Nunez, & Park, 2004). This coherence measure can vary from 0.0 to 1.0 and has been previously used by our laboratory to investigate patterns of functional connectivity associated with cognitive tasks such as mental rotation (Silberstein, 2006; Silberstein, Danieli, & Nunez, 2003) as well as performance on Raven's Advanced Progressive Matrices (Silberstein et al., 2004). As described in our earlier papers, we interpret the SSVEP‐ERPC as a measure of functional connectivity and the terms, SSVEP‐ERPC and FC and will be used interchangeably throughout this article.

In our earlier article (Silberstein et al., 2016), the ADHD and control groups performed the A‐X version of the Continuous Performance Task (CPT A‐X) and a low demand reference task matched for motor components. The control group exhibited high levels of frontoparietal FC during the interval preceding a motor response in the control task and this FC increase was suppressed in the equivalent preparatory interval (the blank interval between the “A” and “X”, or the *A‐X blank*) of the CPT A‐X task. The magnitude of the FC component during the A‐X blank interval of the CPT A‐X task was positively correlated with individual reaction time in the CPT A‐X task, that is to say, participants exhibiting higher levels of FC were slower on the task. The fact that this FC component was reduced during the A‐X blank interval of the CPT A‐X and also positively correlated with reaction is similar to the behavior of the DMN and we suggested that this FC component reflected DMN activity.

While frontoparietal FC was reduced in the A‐X blank interval of the CPT A‐X task in the control group, we observed very different behavior in the ADHD group. Rather than being attenuated in the preparatory interval of the more demanding CPT A‐X task, this FC component was enhanced in ADHD boys during this interval. In summary, our findings suggested that ADHD boys exhibited increased DMN activity in the A‐X blank interval of the more demanding CPT A‐X.

In this study, we examined the FC effects of MPH in drug‐naïve boys newly diagnosed with ADHD while performing the CPT A‐X before and after the administration of MPH.

Two hypotheses are proposed:

1. The administration of MPH will reduce the frontoparietal FC observed in the A‐X blank interval of the CPT A‐X task.
2. The MPH reduction in frontoparietal FC during the preparatory interval will be associated with faster responses on the CPT A‐X task.

## 2 Materials and Methods

### 2.1 Participants

The participants comprised 42 stimulant drug‐naïve males newly diagnosed with ADHD. The mean age of the group was 10.04 years (SD = 2.00 years) and the mean IQ was 107.62 (SD = 9.48). These participants were found to meet eight or more DSM‐IV criteria for ADHD and were newly diagnosed. All ADHD participants were recruited through the Royal Children's Hospital, Melbourne while control participants were recruited through advertisements placed in the wider community. While not the focus of this study, we also include data from a control group for illustrative purposes. The control group comprised 25 males with a mean age of 10.83 years (SD = 1.74 years) and a mean IQ of 110.96 (SD = 6.02).

The study was approved by the Human Research Ethics Committees of Swinburne University, the Royal Children's Hospital and the Australian National Health and Medical Research Council Twin Registry.

### 2.2 Procedures

All participants first performed a low‐demand visual vigilance task which served as a reference task followed by the CPT A‐X task. Both the reference and CPT A‐X tasks were undertaken before and 90 min after the participants were administered their first MPH dose. The dose administered was set at 0.3 mg of MPH per kg of participant weight.

In the reference task, participants viewed a repeated presentation of the numbers 1, 2, 3, 4, and 5 and were required to press a microswitch on the appearance of the 5. In the CPT A‐X task, participants were required to respond on the unpredictable appearance of an X that had been preceded by an A. In all tasks, the numbers remained on the screen for 2 s and were followed by a blank screen for 1.5 s. The ratio of targets to non‐targets was 1:4 and the task duration was 280 s. Reaction time was recorded to an accuracy of 1 millisecond. For all tasks, a correct response to a target was defined as one that occurred no less than 100 ms and no more than 1.5 s after the appearance of the target (5 or an X preceded by an A). Any responses outside the “correct” time intervals were defined as errors of commission, or false alarms, while failure to respond in the correct interval was defined as an error of omission.

The cognitive tasks were presented on a computer monitor. Each letter subtended a horizontal and vertical angle of approximately 1.0° when viewed by subjects from a fixed distance of 1.3 m. The stimulus used to evoke the SSVEP was a spatially diffuse 13‐Hz sinusoidal flicker subtending a horizontal angle of 160° and a vertical angle of 90°, which was superimposed on the visual fields. This flicker was present throughout the task and special goggles enabled subjects to simultaneously view the cognitive task and the sinusoidal flicker.

### 2.3 SSVEP recording/processing

Brain electrical activity was recorded from 64 scalp sites that included all international 10–20 positions, with additional sites located midway between 10 and 20 locations. The specific locations of the recording sites have been previously described (Silberstein, 2006). The average potential of both earlobes served as a reference and a nose electrode served as a ground. Brain electrical activity was amplified and band‐pass filtered (3 dB down at 0.1 Hz and 30 Hz) before digitization to 16‐bit accuracy at a rate of 400 Hz. The major features of the signal processing have been described (Silberstein, 2006; Silberstein et al., 2003). Briefly, the SSVEP was determined from the 13‐Hz Fourier coefficients evaluated over 10 stimulus cycles at the stimulus frequency of 13 Hz, thus yielding a temporal resolution of 0.77 s. The 10‐cycle evaluation period was shifted 1 stimulus cycle and the coefficients were recalculated for this overlapping period. This process was continued until the entire 280 s of activity was analyzed. An identical procedure was applied to data recorded from all 64 recording sites.

### 2.4 Functional connectivity and SSVEP event‐related partial coherence

For each subject, the SSVEP Event Related Partial Coherence (SSVEP‐ERPC) was calculated for all 2016 distinct pairs of electrodes averaged across all correct responses in the reference and CPT A‐X tasks before and after MPH administration.

For the reference task, SSVEP‐ERPC was determined during the 7.0 s interval that comprised a 1.5 s blank period followed by a 2.0 s interval where the number “4” was displayed, as well as the following 1.5 s blank and a 2.0 s interval where the target number “5” was displayed. The equivalent SSVEP‐ERPC was calculated for the “blank”, “A”, “blank”, “X” intervals of the CPT A‐X. Only SSVEP‐ERPC data associated with correct reference and CPT A‐X trials was used. To calculate SSVEP‐ERPC, we used a modified version of the event‐related coherence technique (Silberstein et al., 2003). Partial coherence varies between 0 and 1 and like coherence, is a normalized quantity that is not determined by the SSVEP amplitude at either electrode site. Electrode pairs with high partial coherence indicate relatively stable SSVEP phase differences between electrode pairs across trials. This occurs even though SSVEP phase differences between each of the electrodes and the stimulus may be variable across trials and is equivalent to the removal of the common contribution from the SSVEP stimulus. This means that high SSVEP‐ERPC between electrodes reflects a consistent synchronization between electrodes at the stimulus frequency and is not simply a consequence of two unrelated regions increasing their response to the common visual flicker. Such synchronization reflected in the SSVEP‐ERPC is thought to reflect functional connectivity between the relevant regions and we will use the terms “SSVEP‐ERPC” and “functional connectivity” (FC) interchangeably.

To examine the effects of MPH on FC we considered the following comparisons:

1. FC during pre‐MPH CPT A‐X task with FC during pre‐MPH mean reference task.
2. FC during post‐MPH CPT A‐X task with FC during pre‐MPH mean reference task.
3. FC during post‐MPH CPT A‐X task with FC during pre‐MPH CPT A‐X task.

We used a paired student's *t* test to make the above mentioned comparisons where the *t* test was applied to each point in time for all of the 2016 electrode pairs. To determine the variations in the statistical strength of the differences in FC, we then calculated the number of electrode pairs where the magnitude of student's‐*t* |*t*| is equal to or exceeds a specified threshold level. In this study, we set the threshold |*t*| value for the ADHD group to 3.55 or greater corresponding to *p* ≤ .001. A plot of the number of electrode pairs where the |*t*| exceeds the threshold t value is termed a *Student's t‐frequency curve*. In cases where the number of *t* tests exceeding the threshold of *p* < .01 is so large that the illustration of electrode pairs makes it difficult to identify the patterns of functional connectivity, we use a more demanding criterion corresponding to *p* < .0001.

A permutation test, described in more detail in Silberstein et al., (2016) was used to determine the statistical significance of the number of comparisons where the relevant |t| threshold value was exceeded. This was determined separately for the positive and negative student's *t* values at each of the points in time illustrated in Figs 2, 3, 4, 5, 6. It should be noted that this estimation of the Critical Number of comparisons takes into account the correlation between electrode pairs. The statistical significance associated with the permutation test is indicated by the number of asterisks where 1, 2, 3, and 4 asterisk correspond, respectively, to *p < .01, **p < .005, ***p < .001, and ****p ≤ .0005.

To examine the relationship between MPH‐induced changes in FC during the CPT A‐X task and the MPH‐induced changes in CPT A‐X reaction time (RT), we calculated the linear correlation between the MPH‐induced differences in FC with the corresponding differences in RT. This was done for each point in time yielding 2016 correlation coefficient time series, one for each electrode pair.

To explore temporal variation in the strength of the correlation between the MPH‐induced change in FC (ΔFC) and the corresponding change in RT (ΔRT), we determined the number of electrode pairs where the magnitude of the correlation coefficient r exceeds 0.39, (|*r*|≥0.39) a threshold value corresponding to *p* = .01 at each point in time. Plots illustrating the temporal variation in the number of functional connectivity measures correlated with RT exceeding the threshold are termed “correlation frequency curves”. As with the student's‐*t* frequency curve, a permutation test was used to determine the statistical significance of the number of correlations between RT and FC that exceeded the correlation coefficient threshold *r* value corresponding to *p* ≤ .01. Here, the statistical significance of the permutation test is indicated by the number of asterisks as described above.

## 3 Results

### 3.1 Behavioral data

The CPT A‐X RT for participants prior to and after the administration of MPH is listed in Table 1. We also include RT data for a control group. While the control group data does not address the hypotheses outlined in the introduction, RT and FC data from the control group is included for comparative purposes. The control group data are described in detail in Silberstein et al., 2016.

**Table 1:** Reaction times (RT) (mean and standard deviation) for correct responses while attention deficit hyperactivity disorder (ADHD) and control groups undertook the continuous performance task A‐X task

	Pre‐MPH reaction time	Post‐MPH reaction time	Mean difference	Statistical significance
Control Group	Mean 495 ms, SD = 140 ms	—	—	—
ADHD Group	Mean 570 ms, SD = 142 ms	Mean 569 ms, SD = 140 ms	1.0 ms SD = 140 ms	Paired *t* test Df = 40, *p* = .47
Statistical Significance	Unpaired *t* test Df = 65, *p* = .041			

For the ADHD group, data are presented for reaction times in the session before the administration of methylphenidate (Pre‐MPH) and the session after methylphenidate administration (Post‐MPH). Comparisons indicated that the control groups were faster than the ADHD group and that methylphenidate had no significant effect on mean RT.

As reported in our previous paper (Silberstein et al., 2016), the control group RT is significantly faster than that of the IQ and age‐matched ADHD group. However, when considering the effects of MPH on RT for the ADHD group, we see that there is virtually no effect on the mean RT, see Table 1.

### 3.2 Effects of MPH on functional connectivity

**Figure 1:**
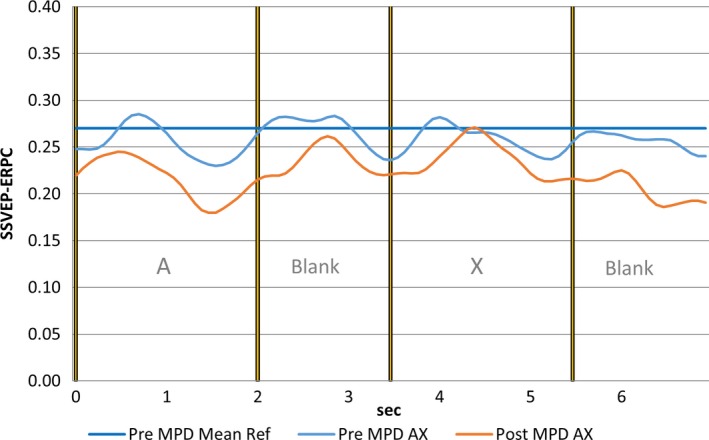
Functional connectivity (FC) during continuous performance task A‐X task for electrode pair Fp2‐Fc5 for the pre‐methylphenidate (pre‐MPH) condition (light blue trace), post‐methylphenidate (post‐MPH) condition (red trace) and the time averaged FC of the pre‐MPH reference task. Note the reduction in FC in the post‐MPH condition

**Figure 2:**
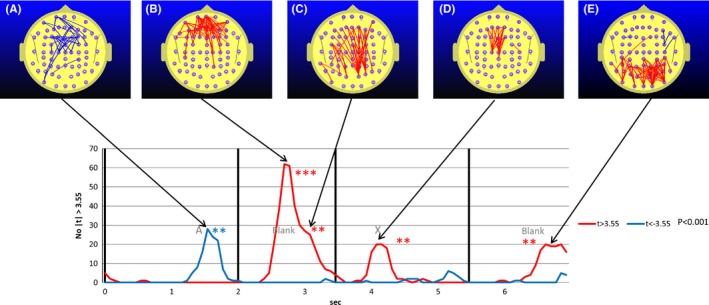
Student's *t*‐frequency graph illustrating the number of electrode pairs where the Student's *t* test comparing attention deficit hyperactivity disorder group functional connectivity (FC) during the continuous performance task A‐X task with the mean of the reference task (meanRef) yielded a difference significant at the *p* ≤ .001 (|*t*|≥2.55) level. Red trace indicates the number of electrode pairs where the student's *t* test indicates that the FC during the reference task is larger than positive meanRef at the *p* ≤ .001 level (t ≥ 3.55). The blue trace indicates the corresponding number of electrode pairs where the student's *t* test indicates that the FC during the reference task is smaller than negative meanRef at the *p* ≤ .001 level (*t* ≤ −3.55). Figs 2A, 2B, 2C, 2D, and 2E illustrate the distribution of electrode pairs corresponding to the points in time associated with peak values in either the red or blue traces. The red lines in Figs 2A to 2E indicate the electrode pairs where FC during the reference task is significantly above that of meanRef (*p* ≤ .001) and the blue lines the equivalent where FC during the reference task is significantly below that of meanRef. Maps are provided for points in time where the permutation test indicates that the number of student's *t* tests exceeding the *t* > 3.55 condition is statistically significant at the *p* ≤ .01 level. Separate permutation tests are carried out for the number of electrode pairs where *t* > 3.55 and *t* < −3.55. The number of asterisks indicates the statistical significance of the number of comparisons that are equal to or exceed the nominated student's *t* threshold value of |*t*|≥3.55 as determined using the permutation test. The blue asterisks indicate the significance of the number for the condition *t* ≤ −3.55, that is when FC in the reference task is less than meanRef while the red asterisks indicate the significance for the number of FC measurements exceeding meanRef at *t* ≥ 3.55. *p ≤ .01, **p ≤ .005, ***p ≤ .001, ****p ≤ .0005. The relationship between the number and color of asterisks and the statistical significance of the number comparisons exceeding the nominated criterion is the same for all subsequent figures

We observed a robust effect of MPH on FC. Figure 1 illustrates the time course of FC for a single electrode pair Fp2 – FC5 while ADHD participants undertook the CPT A‐X task before and after MPH administration. The effect of the MPH is to cause a statistically strong reduction in FC during almost all of the A – X sequence. Of course, this only represents one electrode pair out of the 2016. Figure 2 illustrates the ADHD group student's frequency curve of the number of electrode pairs where the FC difference between the A‐Blank‐X‐Blank interval of the CPT A‐X task and the mean of the reference task (*meanRef*) during the pre‐MPH satisfies the condition |*t*| ≥ 3.55 corresponding to *p* < .001.

**Figure 3:**
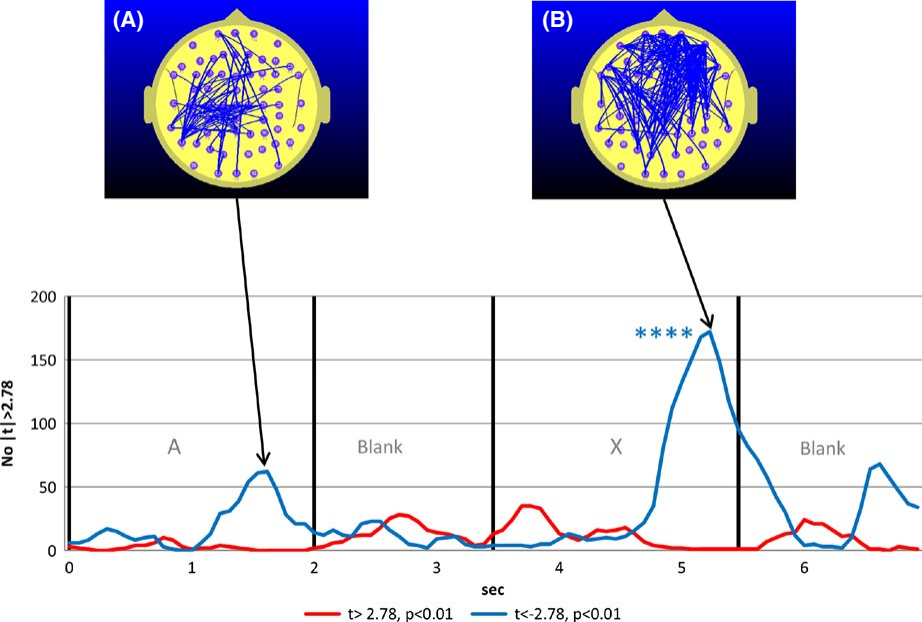
Student's t‐frequency graph illustrating the number of electrode pairs where the student's t test comparing FC during the CPT A‐X task with the mean of the reference task (meanRef) yielded a difference significant at the p<0.01 (|t|>2.78) level in a drug free control group

What is especially striking in Fig. 2 is the increased prefrontal and subsequently frontoparietal FC during the A‐X blank interval. This increase is not apparent in the control group and we reproduce the equivalent student's t frequency curve (Silberstein et al., 2016) for the control group in Fig. 3 for comparison.

**Figure 4:**
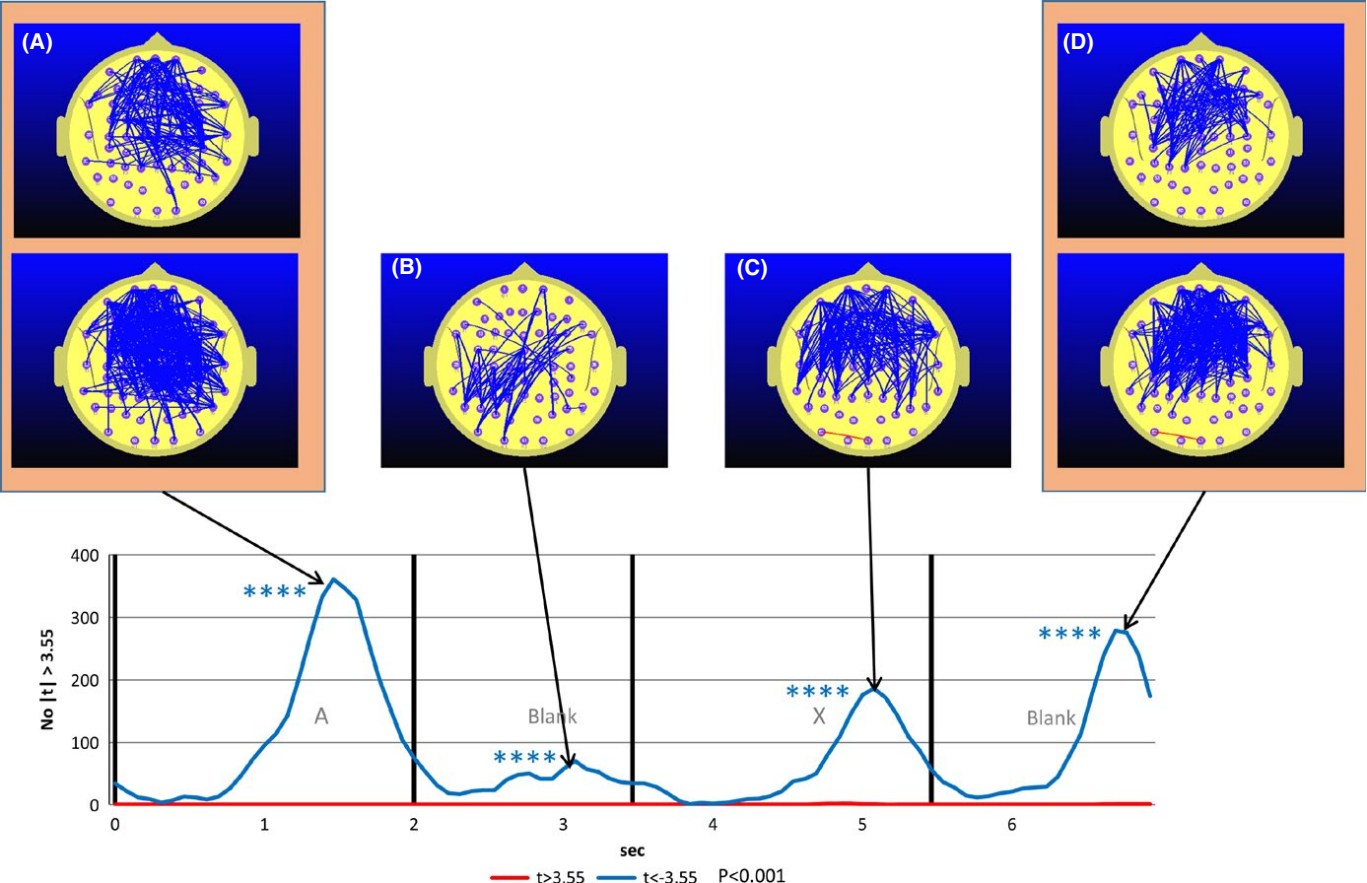
Student's *t*‐frequency graph illustrating the number of electrode pairs where the Student's *t* test comparing attention deficit hyperactivity disorder group functional connectivity during the post‐methylphenidate (MPH) continuous performance task A‐X task with the mean of the pre‐MPH reference task (meanRef) yielded a difference significant at the *p* ≤ .001 (|*t*|≥3.55) level. The upper figures in Fig 4A and 4D illustrate the difference using a more stringent probability threshold (*p* < .0001) to give a clearer indication of the topography of the MPH‐related functional connectivity (FC) decrease. Note that the dramatic increase seen in the pre‐MPH A‐X blank period (Fig. 3B and C) has now been replaced by FC decreases (Fig. 4B)

Figure 4 illustrates the equivalent situation to that illustrated in Fig. 2 for the post‐MPH condition. What is striking here is the complete disappearance of prefrontal and frontoparietal FC increase during the blank period between the A and X. In addition, the FC reductions seen during the A (app 1.5 s) and X (app 5.0 s) interval in the pre‐MPH condition (Fig. 3) are now much larger in the post‐MPH condition (Fig. 4).

**Figure 5:**
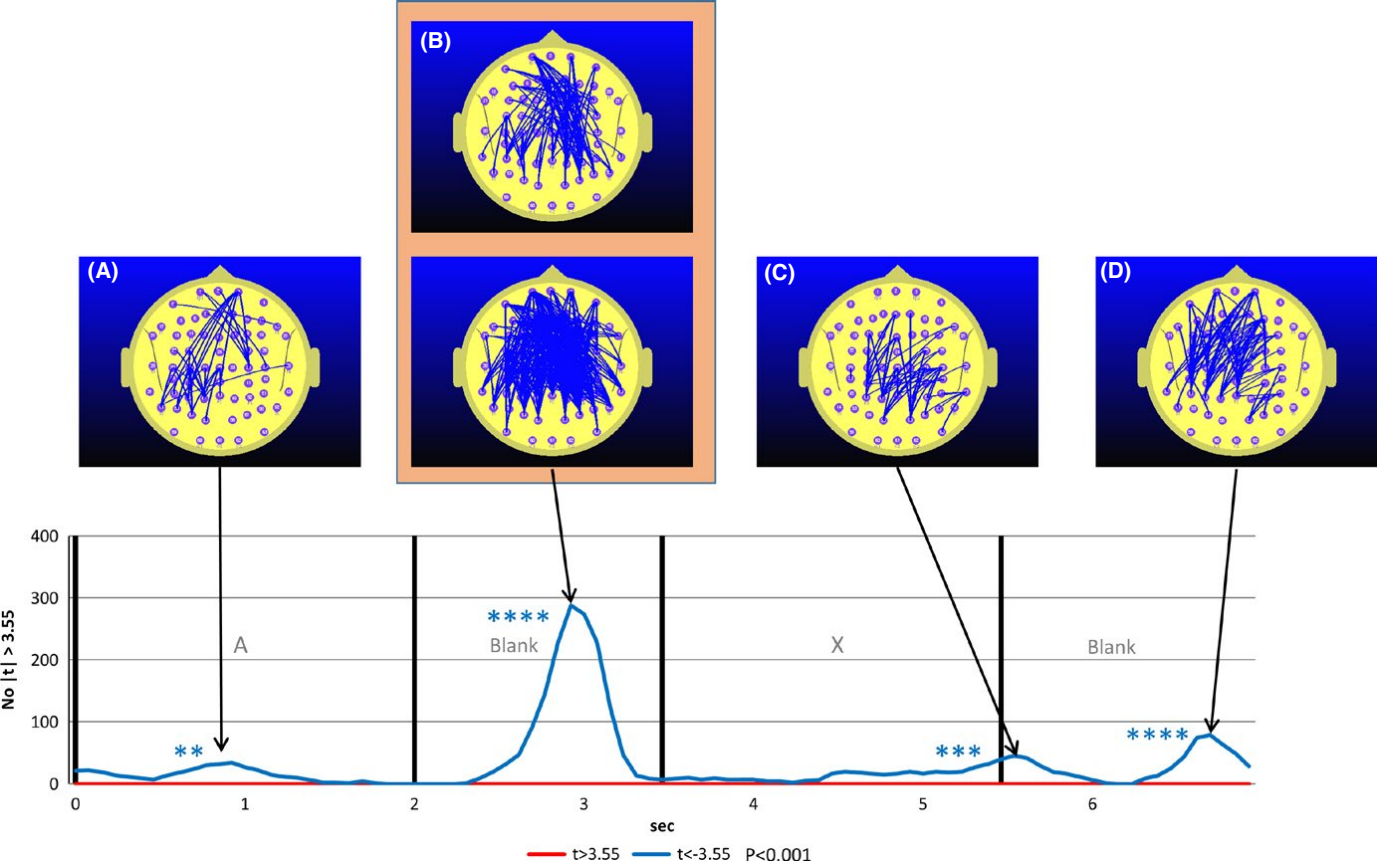
Student's *t*‐frequency graph illustrating the number of electrode pairs where the Student's *t* test comparing attention deficit hyperactivity disorder group functional connectivity (FC) during the post‐methylphenidate (MPH) continuous performance task (CPT) A‐X task with the pre‐MPH CPT A‐X task yielded a difference significant at the *p* ≤ .001 (|*t*|≥3.55) level. The upper figures in Fig 5B illustrates the difference using a more stringent probability threshold (*p* < .0001) to give a clearer indication of the topography of the MPH‐related FC decrease. The most statistically robust effect of MPH on FC is observed in the A‐X blank interval (Fig 5B) where MPH reverses the FC increase observed in the pre‐MPH condition

Figure 5 illustrates the student's t frequency of the number of electrode pairs where the difference between Post‐MPH CPT A‐X FC and Pre‐MPH CPT A‐X FC satisfies the condition |*t*| ≥ 3.55 corresponding to *p* < .001. The most prominent effect of MPH on FC is apparent in the blank interval between the A and X. In this case, the effect of MPH is to reduce frontoparietal FC. As the effect is so strong, the upper map in Fig. 5B illustrates the number of electrode pairs for the more stringent condition where |*t*| < −4.3 corresponding to *p* ≤ .0001.

### 3.3 Effects of MPH on the relationship between FC and reaction time

While there was no difference in the mean RT of the ADHD group before and after the administration of MPH, the standard deviation of the individual RT differences indicates that there was considerable individual variation in the effects of MPH on RT. The largest RT reduction following MPH was 338 ms while the largest RT increase observed was 194 ms.

**Figure 6:**
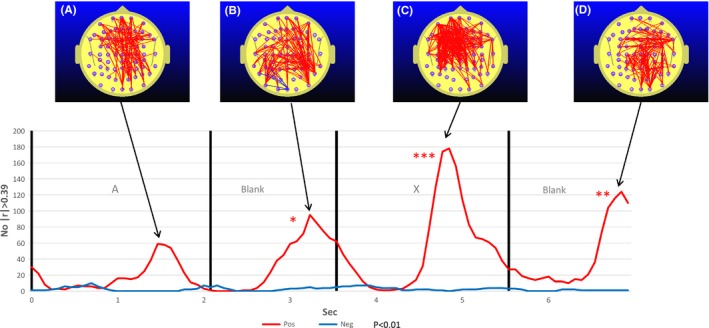
The correlation frequency curve for the number of electrode pairs where methylphenidate (MPH) induced reaction time (RT) differences (postMPH RT – preMPH RT or ΔRT) is correlated with MPH‐induced functional connectivity (FC) differences (postMPH FC—preMPH FC or ΔFC) for |*r*|>0.39, *p* ≤ .01. Red trace indicates the number of electrode pairs where ΔFC is positively correlated with ΔRT, that is, higher ΔFC is associated with a slower individual response. The blue trace refers to the number of electrode pairs where ΔFC is negatively correlated with ΔRT, that is, higher ΔFC is associated with a faster individual response. Overwhelmingly we see a positive correlation, indicating that an MPH‐induced reduction in FC is associated with faster responses in the post‐MPH condition. Fig. 6A is included as the peak number of correlations at this point in time is significant at the 5% level but does not reach the 1% associated with an asterisk

Figure 6 illustrates the correlation frequency curve for the number of electrode pairs where MPH‐induced RT differences (postMPH RT – preMPH RT or ΔRT) is correlated with MPH‐induced FC differences (postMPH FC – preMPH FC or ΔFC). What is striking is the uniformity of the positive correlation during all aspects of the 7 s segment. In all of the segments, higher FC was correlated with slower individual responses. The other point to note is that each peak in the correlation frequency curve coincides with a peak in the student's *t* frequency curve illustrated in Fig. 4. In addition, the correlation topography illustrated in Fig. 6A and C and to a lesser extent Fig. 6B are similar to the FC topography illustrated in Fig. 4A and C and to a lesser extent Fig. 4B. The comparison of Figs 6 and 4 indicates that MPH reduces FC (Fig. 4) and that the greater the MPH‐induced FC reduction, the faster the mean individual response.

## 4 Discussion

Our findings indicate that MPH had a profound effect on FC while participants performed the CPT A‐X task. When comparing the post‐MPH versus pre‐MPH FC, the largest effect was apparent in the A‐X blank period. Specifically, MPH dramatically reduced the frontoparietal FC increase observed in the pre‐MPH A‐X blank interval rendering the ADHD FC changes similar to those seen in the control group. As such, we suggest that the findings confirm the first hypothesis. In our earlier paper (Silberstein et al., 2016), we presented evidence that the frontoparietal FC component observed in the A‐X blank interval was an indication of DMN activation. Our current findings concerning the effects of MPH on this FC component observed during the A‐X blank interval confirm our suggestion that this component reflects DMN activity.

More generally, the interpretation of our findings that MPH suppresses the DMN is consistent with a number of studies examining the effects of stimulants on the DMN. In addition to the studies cited in the introduction a number of fMRI, magnetoencephalography (MEG) and EEG studies provide evidence consistent with the notion that MPH reduces DMN activity. In an MEG study, Franzen et al. (2013) observed that ultra‐low frequency (0.2–2.0 Hz) coherence between left and right inferior parietal lobes was elevated in a group of adults diagnosed with ADHD compared to the adult control group. This ultra‐low frequency component has been identified as an index of DMN activity (Helps et al., 2010). Franzen et al. (2013) reported that this component was reduced in response to the administration of dextroamphetamine, a dopamine reuptake blocker. Similar findings were reported by Cooper et al. (2014) in an EEG study examining the effects of MPH in a group of adults diagnosed with ADHD while performing a CPT task with flankers. Compared to controls, the ADHD group exhibited increased very low frequency EEG (0.02–0.2 Hz) power at parietal sites that was correlated with errors of omission. MPH was found to reduce the very low frequency EEG component.

### 4.1 Methylphenidate‐induced differences in RT are correlated with FC changes

While MPH did not appear to have an effect on the group mean RT, this group finding obscured significant individual RT differences due to MPH. Importantly, those participants who exhibited the greatest RT reduction were the one that showed the greatest MPH‐related frontoparietal FC or DMN activity reduction. In addition, the points in time where MPH reductions of FC (with respect to pre‐MPH baseline) were greatest (see Fig. 4) are precisely those points in time where changes in FC are most strongly correlated with changes in RT (see Fig. 6). Furthermore, the peak correlation between RT difference and FC difference occurring during the A‐X blank is characterized by a parietofrontal topography (Fig. 6B) similar to that occurring around this time in the pre‐MPH FC seen in Fig. 3C. This reinforces the indication that it is the ADHD frontoparietal FC increase observed in the A‐X blank that is correlated with reaction time and thus more likely an indication of DMN activity. This relatively robust correlation between frontoparietal FC and RT also confirms our second hypothesis that predicted a positive correlation between MPH‐induced changes in FC and the corresponding changes in RT.

Our data also suggest that individual variation in the effect of MPH on the DMN is reflected in variations in RT. This may in part account for some discrepancies in the reported effects of MPH on RT in the CPT. For example, Michael, Klorman, Salzman, Borgstedt, and Dainer (1981) and Klorman et al. (1983) reported reduced RT after MPH intake, while Satterfield and Cantwell (1974) reported an RT increase and Lawrence et al. (2005) no change in RT. While variations in the manner in which the CPT was administered may account for some of these mean RT differences, another important factor may involve genetic differences in the dopaminergic and noradrenergic systems (Bellgrove et al., 2005; Cook et al., 1995; Cummins et al., 2012; Gilbert et al., 2006; Froehlich et al., 2011).

### 4.2 The relevance of top‐down and bottom‐up processes in cortical communication

Our findings of reduced FC in response to MPH appear to be consistent with previous observations pointing to MPH and other dopaminergic reuptake blockers reducing DMN activity. However there does appear to be an inconsistency in that some fMRI studies have also reported evidence of increased FC in response to MPH. For example, MPH intake has been associated with increases in frontoparietal FC when ADHD diagnosed participants perform a CPT (Rubia et al., 2009) or a Sternberg working memory task (Wong & Stevens, 2012). However, our findings illustrated in Figs 5 and 6 give no indication of any increases in FC following MPH and one may ask *why don't we observe some of the FC increases associated with MPH that have been reported in fMRI studies*? The following discussion section considers the factors that might account for this apparent inconsistency.

There are a number of factors that could contribute to the disparity between our observations of MPH‐induced FC changes and those reported using fMRI. One obvious factor may be the differences in time scale between SSVEP and fMRI indications of FC increases. The SSVEP is clearly able to register far more rapid changes in FC than fMRI (Nunez & Silberstein, 2000). Furthermore, the neural processes that are most readily apparent using fMRI‐ and EEG‐based systems such as SST may be quite different (Nunez & Silberstein, 2000). However, we consider it unlikely this constitutes the main explanation for the apparent absence of MPH‐induced FC increases.

We suggest another factor that is specific to the manner in which SSVEP is utilized in this study may play a crucial role in accounting for this disparity. To appreciate this, we note the extensive evidence for communication between cortical regions being mediated by synchronous oscillations. We refer readers to the excellent review by Fries (2015). More recently, it has been appreciated that different frequency components of the brain activity make different types of contributions to information transfer between different cortical regions or cortico‐cortico communication. Early work by Von Stein, Chiang, and König (2000) indicated that short‐range monosynaptic interactions were mediated preferentially by activity in the gamma frequency range (30–90 Hz) while longer range polysynaptic interactions were mediated by lower frequency activity in the alpha (8–12 Hz) and beta (13–25 Hz) range. Subsequently, this picture has been clarified further and to appreciate this, we need to introduce the concepts of *bottom‐up* and *top‐down* processes.

These concepts are best illustrated in the functioning of the visual cortex. It is now appreciated that the various cortical regions mediating visual processing are organized in a hierarchic fashion with lower region such as the primary visual cortex (Brodmann area 17) receiving its input from the lateral geniculate nucleus of the thalamus and projecting up the hierarchy (feed‐forward) to one of the visual association areas (Brodmann area 18). Area 18 in turn projects up to higher visual processing areas such as Brodmann area 19 (Felleman & Van Essen, 1991; Markov et al., 2014). These feed‐forward flow of information are determined primarily by the sensory input and are termed “bottom‐up”. At the same time, all the cortical regions receiving a feed‐forward projection such as Brodmann 18 also give rise to a feedback projection onto the lower region such as Brodmann area 17 and these are termed “top‐down”. Recent studies now indicate that these bottom‐up and top‐down information transfer processes are mediated by synchronous oscillations at different frequency ranges. Bottom‐up processes are mediated primarily by synchronous oscillations in the gamma frequency band and to a lesser extent in the theta band. By contrast, top‐down processes appear to be mediated by synchronous oscillations in high‐alpha and beta frequency range (10–20 Hz) (Bastos et al., 2015; Bressler & Richter, 2015; Buffalo, Fries, Landman, Buschman, & Desimone, 2011; Fries, 2015). In considering these factors, we are now in a position to consider the original question, *why don't we observe some of the FC increases associated with MPH that have been reported in fMRI studies*?

The SSVEP‐ERPC methodology we have used in this study utilizes a continuous 13 HZ visual flicker to elicit the SSVEP that is the basis of the FC measure reported here. Now, 13 Hz is located in the 10–20 Hz frequency band that is used for top‐down processes. Thus, on the basis of the stimulus frequency used, our data are likely to be dominated by top‐down communication and be relatively insensitive to bottom‐up communication. This strong preferential sensitivity for top‐down communication has a number of implications. Firstly, some of the FC increases seen in fMRI studies may be a manifestation of bottom‐up processes mediated at gamma and theta frequency synchronous oscillations. As such, these bottom‐up processes may not be *seen* by the SSVEP‐ERPC methodology when using a 13 Hz visual flicker.

Another important factor that may contribute to the fact that we observe no MPH‐induced FC increases is the position of the DMN in the hierarchy of cortical information processing. Specifically, the DMN is considered at or near the apex of the cortical hierarchy (Bressler & Menon, 2010; Carhart‐Harris & Friston, 2010; Fazelpour & Thompson, 2015). As a network at (or near) the top of the cortical processing hierarchy, almost all of its outputs would be top‐down and these are in turn mediated by synchronous oscillations in the 10–20 Hz range. As such, they would be preferentially detected by our methodology when using the 13 Hz visual flicker to elicit the SSVEP. Furthermore, as the DMN is at the apex of the cortical information processing hierarchy, the top‐down projection of the DMN onto other networks would also mediated by the frequency range that our SSVEP‐ERPC methodology is most sensitive to. In summary, the FC changes we have reported may directly represent FC changes in the DMN as well as indirect effects of the DMN acting on other possibly cortical networks. Some of these indirect effects may well be seen most prominently as FC changes in task‐positive networks. We suggest that this may be another mechanism accounting for the prominent frontoparietal FC changes reported in this and a previous paper (Silberstein et al., 2016).

In this paper, we have interpreted the DA‐induced reductions in FC in terms of the behavior of the DMN. However, dopaminergic processes are apparent throughout the cortex and the effects of DA obviously extend well beyond the DMN. For example, in Parkinson's disease, a disorder associated with the loss of DA in the substantia nigra, we find that motor dysfunction is positively correlated with abnormal alpha and beta frequency functional connectivity between cortical regions as well as between the basal ganglia and cortex. The administration of L‐DOPA, the DA precursor eliminates this abnormal FC which is in turn correlated with reduced motor dysfunction (Williams et al., 2002). More generally, we suggest that DA and nor‐adrenaline (NA) the other catecholamine may play an important role in suppressing unnecessary or irrelevant communication between cortical and subcortical networks necessary for the performance of cognitive or motor tasks. More specifically, it is suggested that the capacity to dynamically suppress or *decouple* functional connectivity may be a critical determinant of cognitive and motor aptitude (Silberstein, 2006).

### 4.3 Concluding comments

In this and the previous study in this series (Silberstein et al., 2016), we observed a transient increase in frontoparietal FC during the A‐X blank of the CPT A‐X task in boys diagnosed with ADHD. Administration of MPH in the ADHD group robustly suppressed this FC increase and partly normalized the FC changes seen in the CPT A‐X task. Furthermore, MPH‐induced changes in RT were positively correlated with MPH‐induced changes in FC revealing that MPH‐induced reductions in transient A‐X blank FC were associated with faster CPT A‐X responses in the post‐MPH condition. The findings outlined here are consistent with the suggestion that the FC changes observed during the A‐X blank interval reflect, directly or indirectly DMN activity.

## Conflict of Interest

None of the authors have a conflict of interest to declare.
